# The immunological and structural epidermal barrier dysfunction and skin microbiome in atopic dermatitis-an update

**DOI:** 10.3389/fmolb.2023.1159404

**Published:** 2023-08-16

**Authors:** Tubanur Çetinarslan, Lisa Kümper, Regina Fölster-Holst

**Affiliations:** ^1^ Department of Dermatology and Venereology, Manisa Celal Bayar University, Manisa, Türkiye; ^2^ MEDICE Arzneimittel Pütter GmbH and Co. KG, Iserlohn, Germany; ^3^ Department of Dermatology-Venereology and Allergology, Universitätsklinikum Schleswig-Holstein, Kiel, Germany

**Keywords:** skin microbiome, atopic dermatitis, keratinocytes, epidermal barrier, filaggrin

## Abstract

Atopic dermatitis (AD) is a common, chronic and relapsing inflammatory skin disease with various clinical presentations and combinations of symptoms. The pathophysiology of AD is complex and multifactorial. There are several factors involved in the etiopathogenesis of AD including structural and immunological epidermal barrier defect, imbalance of the skin microbiome, genetic background and environmental factors. Alterations in structural proteins, lipids, proteases, and their inhibitors, lead to the impairment of the stratum corneum which is associated with the increased skin penetration and transepidermal water loss. The elevated serum immunoglobulin E levels and blood eosinophilia have been shown in the majority of AD patients. Type 2 T-helper cell immune pathway with increased expression of interleukin (IL)-4, IL-5, and IL-13, has an important role in the etiopathogenesis of AD. Both T cells and keratinocytes contribute to epidermal barrier impairment in AD via a dynamic interaction of cytokines and chemokines. The skin microbiome is another factor of relevance in the etiopathogenesis of AD. It has been shown that during AD flares, *Staphylococcus aureus* (*S. aureus*) colonization increased, while *Staphylococcus epidermidis* (*S. epidermidis*) decreased. On the contrary, *S. epidermidis* and species of *Streptococcus*, Corynebacterium and Propionibacterium increased during the remision phases. However, it is not clear whether skin dysbiosis is one of the symptoms or one of the causes of AD. There are several therapeutic options, targeting these pathways which play a critical role in the etiopathogenesis of AD. Although topical steroids are the mainstay of the treatment of AD, new biological therapies including IL-4, IL-13, and IL-31 inhibitors, as well as Janus kinase inhibitors (JAKi), increasingly gain more importance with new advances in the therapy of AD. In this review, we summarize the role of immunological and structural epidermal barrier dysfunction, immune abnormalities, impairment of lipids, filaggrin mutation and skin microbiome in the etiopathogenesis of AD, as well as the therapeutic options for AD and their effects on these abnormalities in AD skin.

## 1 Introduction

Atopic dermatitis (AD) is a common chronic and relapsing skin disease which usually occurs in the first years of life and affects ∼20% of children worldwide (Bylund et al., 2020). The prevalence of AD is increasing. in both children and adults (Fuxench et al., 2019; Lee et al., 2019). AD is characterized by chronic inflammation which is associated with animpaired immunological response and epidermal barrier dysfunction. This chronic inflammation leads to itching in AD patients due to dry skin, mechanical injury and allergic sensitization to environmental antigens (Oyoshi et al., 2009).

In general, AD is associated with other atopic comorbidities such as asthma, rhinitis, conjunctivitis, and food allergy (Paller et al., 2019a; Kim et al., 2019). The acute phase of AD is characterized by erythematous papules and vesicles, accompanied by itching. With the evolution to the chronic phase, lichenified lesions occur as a result of dermal fibrosis. The location of AD lesions varies with the age of the patient. In adults, it predominantly occurs in the skin flexures, face and extremities, while patients under 1 year of age generally present widely distributed lesions. The cheeks are usually the first affected area in infantil period (Bieber, 2022).

**FIGURE 1:**
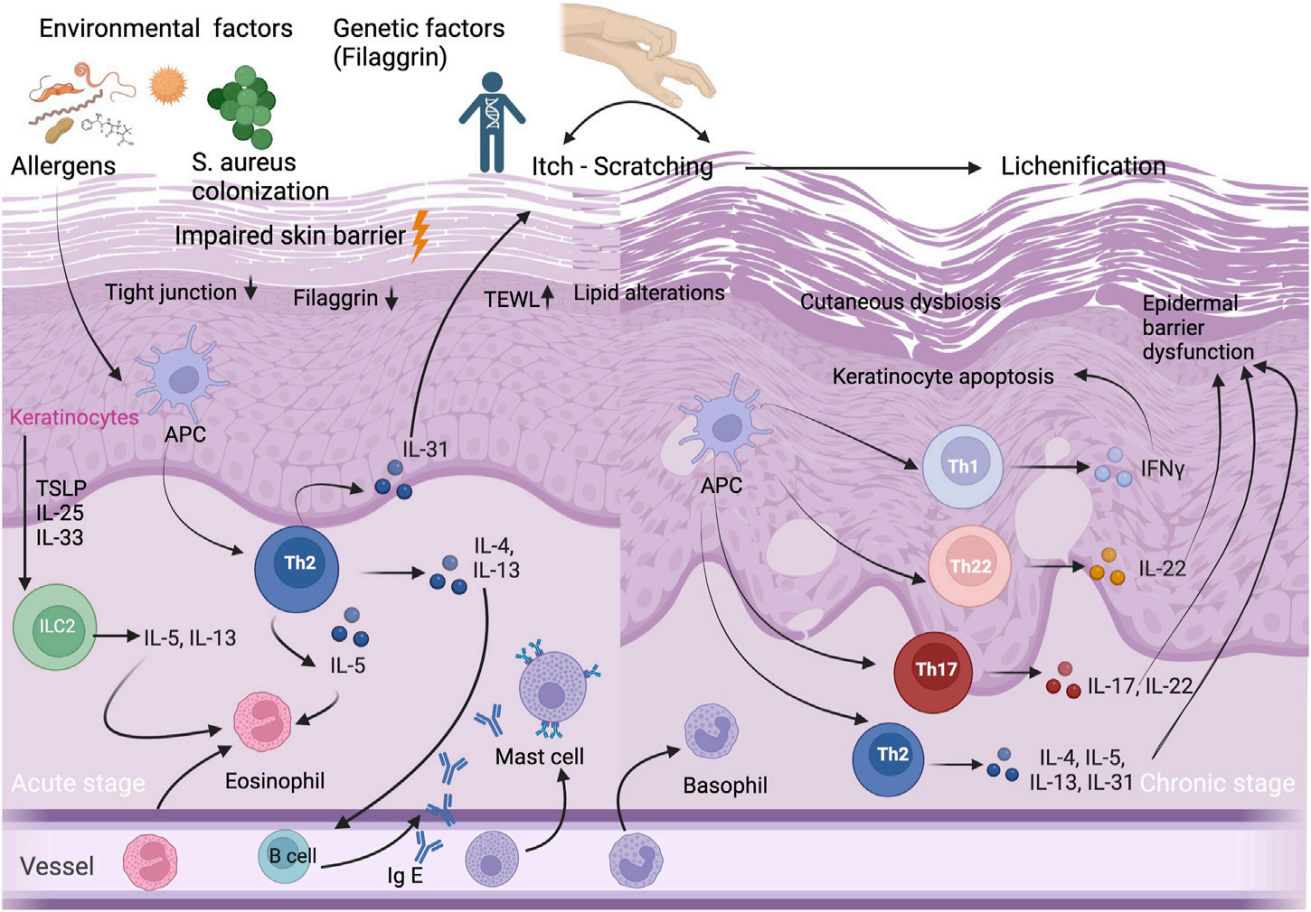
Immunopathogenesis of AD. Acute and chronic stages of AD and selected T-cell subpopulations and interleukins, other inflammatory cytokines and IgE antibodies, and selected cell populations ILC2, APCs, and eosinophils, mast cells, basophils, eosinophils and keratinocytes that play an important role in AD etiopathogenesis. Impaired skin barrier is associated with decrease in filaggrin protein, increase in TEWL, lipid alterations and cutaneous dysbiosis. Abbreviations: APC, antigen presenting cell; IFN, interferon; Ig E, immunoglobulin E; IL, interleukin; ILC2, innate lymphoid cell 2; S.aureus, *Staphylococcus aureus*; TEWL, transepidermal water loss; Th, T helper; TSLP, thymic stromal lymphopoietin cell.

The most important factors in the etiopathogenesis of AD are genetic background, imbalance of the skin microbiome, environmental factors, as well as structural and immunological epidermal barrier defect which leads to transepidermal water loss (TEWL) (Figure 1). The increased TEWL which is associated with increased permeability of the stratum corneum (SC), is a characteristic finding of both lesional and non lesional skin of AD patients (Dizon et al., 2018). The magnitude of increase in TEWL is also correlated with the disease severity (Dizon et al., 2018). Dry skin which is one of the hallmarks of AD, occurs due to increased water loss and it leads to pruritus that impairs AD patients’ quality of life (Goerdt et al., 1999).

This review will focus on the immunological and structural epidermal barrier dysfunction in AD and the role of keratinocytes, filaggrin (FLG) mutation, lipid alterations in SC, as well as skin microbiome in AD and the therapeutic options in the treatment of AD and their effects on AD skin.

### The immune abnormalities in AD and their contribution to epidermal barrier dysfunction

AD is a complex, multifactorial disease in which both innate and adaptive immune system contribute to its etiopathogenesis (Figure 1). Previously, it has been thought that AD is simply a Th2-mediated inflammatory disease since the majority of patients have increased serum immunoglobulin E (IgE) levels and high numbers of circulating eosinophils (Tokura and Hayano, 2022). However, it has been demonstrated that there is a biphasic switch from Th2 to Th1 responses in both acute and chronic skin lesions of AD patients (Leung, 2000). Moreover, we know that the immunological pathway of AD is not simple and also characterized by a dysfunction in the immune system, with a dominant Th2/Th22 skewing, and variable activation of Th17/Th1 subtypes (Tokura and Hayano, 2022). In chronic AD lesions, it has been reported that there is a complex inflammation pathway between T helper cells (Th1/Th2/Th17/Th22) and hyperproliferative keratinocytes which are characterized by altered terminal differentiation (Renert-Yuval et al., 2021).

IL-4 and IL-13 are two major cytokines in the etiopathogenesis of AD and play a critical role in the differentiation of Th2 cells and the production of IgE. The increased skin expression of Th2 cytokines including IL-4, IL-5, and IL-13, has been shown in acute AD skin lesions (Oyoshi et al., 2009). These cytokines stimulate IgE antibodies and eosinophils in both skin and peripheral blood (Matsunaga and Yamauchi, 2016). IL-4 and IL-13 disrupt the epidermal barrier integrity by decrease of main terminal differentiation proteins including filaggrin, loricrin, and involucrin (Cork et al., 2006).

Additionally, IL-4 decreases the expression of genes in the epidermal differentiation complex in keratinocytes and it leads to epidermal barrier dysfunction and impaired innate skin immune system, and consequently to an increased risk of infections (Sehra et al., 2010). IL-13 overexpression has been found in both lesional and non-lesional skin of AD patients (Tsoi et al., 2019) and its levels correlate with AD disease severity (Szegedi et al., 2015; Guttman-Yassky et al., 2019; Tsoi et al., 2019). Moreover, it has been reported that IL-13 messenger RNA and protein levels are higher compared to IL-4, although both cytokines play a major role in the pathogenesis of AD (Tsoi et al., 2019). This highlights the importance of IL-13 inhibition in AD therapy.

The impairment of the epidermis due to various factors such as infectious agents, allergens or mechanical trauma may stimulate inflammation processes and leads to production of proinflammatory mediators such as TSLP (Thymic stromal lymphopoietin), which is an IL-7–like cytokine, IL-4, IL-13, IL-25, and IL-33 (Camelo et al., 2017; Klonowska et al., 2018). Thus, these increased inflammatory cytokines induce immune cell accumulation, which leads to formation of nitrogen oxide and reactive oxygen species (Mittal et al., 2014).

Various antimicrobial peptides play an important role in the innate immunity (Lehrer et al., 1993). The expression of human *β*-defensin 2 (hBD-2) and LL-37, is triggered by the inflammation of the skin (Frohm et al., 1997). In some AD patients, it has been reported that the decreased antimicrobial and immunomodulatory peptides such as LL-37, hBD-2, and hBD-3, are associated with skin infections (Niyonsaba et al., 2017; Nguyen et al., 2020). Host defense peptides (HDPs) induce cytokine and chemokine production and promote cell proliferation and migration (Reinholz et al., 2012; Pahar et al., 2020), and also contribute to normal epidermal barrier function against TEWL via organizing the distrubiton of tight junction protiens (Akiyama et al., 2014). The predominance of Th2-related cytokines, which is associated with an inhibitory effect against LL-37, hBD-2, and hBD-3 production, might be one of the reasons for reduced HDP levels in AD patients (Reinholz et al., 2012; Akiyama et al., 2014; Nguyen et al., 2020).

### Keratinocytes play a major role in immunological and structural epidermal barrier impairment in AD

The human epidermis is a unique protective barrier against transcutaneous water loss, penetration of microbial pathogens and ingress of toxins and allergens (Marks, 2004; Elias, 2005). The epidermis consists of four layers: the stratum basale, the stratum spinosum, the stratum granulosum and the SC (Matsui and Amagai, 2015). The SC, which is the outermost layer of skin and the most important part of the epidermal barrier, comprises 20 layers of corneocytes, which are embedded in intercellular lipids (Oyoshi et al., 2009). The impairment of the SC, which protects against environmental factors, allergens and water loss, leads to epidermal barrier dysfunction in AD (Schleimer and Berdnikovs, 2017).

Keratinocytes have a critical role both in the pathophysiology of epidermal barrier defect and in the activation of the innate immune response. They receive both mechanical and inflammatory stimuli, and produce antimicrobial peptides (AMPs) and proinflammatory cytokines. In patients with AD, the epidermis is characterized by a block in terminal keratinocyte differentiation (Guttman-Yassky et al., 2009) which leads to allergen penetration through the epidermis and systemic IgE sensitization (Jensen et al., 2004), and reduced expression of skin barrier proteins including FLG, involucrin, loricrin, as well as AMPs (Ong et al., 2002; Howell et al., 2007). AMPs including LL-37, hBD-2, and hBD-3, play a major role in repairing the impaired epidermal barrier. They also show an autocrine function on keratinocytes, which produce pro-inflammatory cytokines including IL-25, IL-33, and TSLP. This inflammatory cytokines activate the innate lymphoid cell 2 (ILC2), dendritic cells and langerhans cells, and initiate the differentiation of type 2 immune response (Nakatsuji et al., 2017; Radi et al., 2022). ILC2s have an important role in homeostasis and produce a variety of cytokines, primarily IL-5 and IL13 (Mielke et al., 2013). ILC2s which present CD1a, have been found to be increased and activated in AD lesional skin (Hardman et al., 2017) (Figure 1).

TSLP which is secreted by epidermal keratinocytes, also induces other cells such as dendritic cells, T cells, as well as mast cells. Mast cells play an important role in IgE-mediated hypersensitivity and allergic diseases, as well as in AD. It has been shown that mast cells are significantly increased in AD skin (Kawakami et al., 2009). Not only keratinocytes and mast cells, but also T cells have a major role in skin barrier dysfunction via cytokine secretion (Humeau et al., 2022). Kallikreins (KLK) are proteases which have various functions including the induction of pro-inflammatory cytokine production by keratinocytes, the degradation of intercellular adhesion molecules, as well as the regulation of barrier integrity (Briot et al., 2009). In the SC of AD patients, the levels of KLK5 and KLK7 increase by the stimulation of IL-4 and IL-13 (Komatsu et al., 2007). IL-4, IL-13, IL-31, and IL-22 expressed by Th2 and Th22 cells, may disturb epidermal barrier function via scratching due to itch in AD patients (Furue et al., 2018). Although the predominance of type 2 response in AD is well-known, there is a complex pathway, including Th2, Th22, Th17 and Th1 subtypes, associated with regulatory T cell (Treg) dysfunction (Humeau et al., 2022).

In patients with AD, various abnormalities manifest not only in the SC, but also in other epidermal layers. Degradation in the cells of the stratum spinosum and stratum granulosum with a concomitant expansion of cells in the stratum basale has been described (Jensen et al., 2004). Totsuka et al. investigated the changes of structural proteins and adhesion molecules in the stratum spinosum of AD lesional skin, and the effect of Th2 cytokines including IL-4 and IL-13 on expression of these proteins (Totsuka et al., 2017). In AD lesional skin, they found decreased expression of keratin 1, keratin 10, desmoglein 1 and desmocollin 1, which is supressed by Th2 cytokines (Totsuka et al., 2017).

However, it is not certain that the epidermal skin barrier defect in lesional skin of AD is a primary factor or a process due to disease activity. The epidermal barrier abnormality has been reported not only in lesional skin, but also in non-lesional skin of AD patients (Proksch et al., 2006). Moreover, the epithelium of AD, which is not affected by skin lesions, is also characterized by bioelectric abnormalities in tight junctions (Pelc et al., 2018). Tight junctions are intercellular junction units and have an important role in the formation of the epidermal barrier against the transition of water, ions, and macromolecules (Basler et al., 2016). Dysfunction of tight junctions as a result of cutaneous inflammation in AD skin impairs epidermal barrier permeability by altering the pH of the SC which leads disruption of the mature lamellar structures, polar lipid formation and keratohyalin granules (Yuki et al., 2013). As a result, epidermal barrier permeability increases and it leads to increased ease of transition of the various bacterial agents and allergens, leading to a vicious circle of epidermal barrier dysfunction and cutaneous inflammation (Yokouchi et al., 2015; Katsarou et al., 2023).

### Lipid alterations in AD

The epidermis contains basal keratinocytes, which are highly proliferative and have differentiation capability (van Smeden et al., 2014). They are characterized by the organized expression of specific proteins, intercellular junctions, enzymes such as proteases/antiproteases, as well as lipid components (van Smeden et al., 2014). The main SC lipids including ceramides (CERs), free fatty acids (FFAs) and cholesterol and its esters, are essential for a healthy skin barrier (Pappas, 2009; Jungersted and Agner, 2013; Loiseau et al., 2013; Li et al., 2016). SC lipids are secreted by keratinocytes into the extracellular space and move to the stratum corneum via lamellar bodies, which consist mostly of phospholipids, sphingolipids, and cholesterol (Wertz, 2018). In lamellar bodies, these lipids are metabolized by various enzymes such as sphingomyelinase, glucocerebrosidase and phospholipase (Wertz, 2018).

The intercellular lipid membrane shows a barrier role against infectious agents and maintains the integrity of the SC (Elias, 2005). An ideal ratio of the different SC lipids is one of the most important factors for healthy epidermal barrier function. AD is a common dermatological disease, characterized by impaired lipid barrier function. Mutations in lipid metabolizing enzymes or mutations that lead to increased protease activity or decreased protease inhibitor activity, could be associated with epidermal barrier dysfunction in AD. The lipid group most commonly reported to be deficient in AD is CERs (Levin et al., 2013). AD is characterized by abnormal skin lipids that are stimulated by hyperactivated type 2 immune response (Berdyshev et al., 2018). Inflammatory cytokines (IL-4, IL-13 and IL-31) reduce the expression of main CER synthesizing enzymes which are essential for lipid formation (Danso et al., 2017). This abnormality in SC lipids seems to occur independently of FLG mutations (Janssens et al., 2012; Joo et al., 2015). The metabolism of CERs in AD is affected by the immune/inflammatory response. Decreased total CER levels and variety in chain length such as increased short chain CERs, diminished long-chain CERs, increase in short chain FFAs, reduction in long chain FFAs and decrease in hydroxy-FFAs, have been shown in the etiopathogenesis of AD (van Smeden et al., 2014b; van Smeden and Bouwstra, 2016).

The enrichment of the skin microbiota with ceramidase-secreting bacteria, has been reported as a possible cause of reduced levels of CERs in AD skin (Ohnishi et al., 1999). Although *S. aureus* colonization is an important factor in AD patients, *S. aureus* does not show ceramidase activity (Ohnishi et al., 1999). Furthermore, so far no ceramidase-producing bacteria have been found in the skin of patients with AD (Pavel et al., 2022). One alternative hypothesis which seems more reasonable to explain the reduced levels of CERs, is the stimulation by Th2 cytokines such as IL-4 which leads to reduced CER synthesis in keratinocytes (Hatano et al., 2005).

These alterations in lipids lead to increased TEWL in AD skin (Janssens et al., 2012; Meckfessel and Brandt, 2014; Danso et al., 2017). Moreover, the decreased lipid levels are not only limited to lesional skin, but also have been demonstrated in non-lesional skin of patients with AD (van Smeden et al., 2014b; Joo et al., 2015; Toncic et al., 2020). This indicates that AD is not limited to visible skin lesions only.

### The key protein filaggrin in AD

FLG is major epidermal structural protein which is provided by upper-layer epidermal keratinocytes. FLG is produced as a polymer profilaggrin and it is located in the outer nucleated layers of the epidermis. FLG degradation products maintain the pH balance, skin hydration and antimicrobial function of the epidermal barrier (Brown and McLean, 2012; Harding et al., 2013; Drislane and Irvine, 2020; Kim and Lim, 2021). FLG mutations are the most well-known genetic risk factors in AD (Morar et al., 2007; Esparza-Gordillo et al., 2009). The Th2 pathway plays an important role in AD and other atopic comorbidities, such as allergic rhinitis, asthma, and food allergy. Th2-related (IL-4, IL-13, IL-25), and Th22-related (IL-22) cytokines, which lead to reduced FLG levels in keratinocytes, are involved in the etiopathogenesis of AD (Fenner and Silverberg, 2018).

FLG mutations which are the most common, affecting 30%–50% of white AD patients, are associated with an increased skin permeability (Patrick et al., 2021). FLG deficiency is a complex combination of dysregulation of molecules involved in inflammatory, proteolytic and cytoskeletal functions (Elias et al., 2017). FLG deficiency is associated with dry skin, in consequence of the impaired skin barrier function and increased TEWL. This altered epidermal barrier function also makes the skin vulnerable against irritants, haptens and allergens, which penetrate the skin and induce allergic sensitization (Ständer, 2021).

In murine models of AD, it has been shown that FLG deficiency changes the construction of keratinocytes and secretion of lipids (Man et al., 2008; Kawasaki et al., 2012; Thyssen and Kezic, 2014; Elias et al., 2017). The decreased skin lipids lead to the diminished production of epidermal AMPs and consequently an altered skin microbiome (Langan et al., 2020; Patrick et al., 2021). In support of this, in AD patients with FLG mutations, an elevated *S. aureus* colonization has been shown (Clausen et al., 2017). In addition to the imbalance of the skin microbiome, FLG mutation is also associated with higher risk of early onset of the disease, high serum levels of IgE and other manifestations of atopy, as well as the persistence of AD into adulthood (Zaniboni et al., 2016).

Haftek et al., examined biomechanical characteristics of corneocytes in children with AD with FLG mutations (Haftek et al., 2020). In these corneocytes, they showed a decreased elastic modulus which strongly correlated with FLG degradation products and TEWL, but not with SCORAD (SCORing Atopic Dermatitis) (Haftek et al., 2020). They suggested that AD patients have decreased corneocyte stiffness, which correlates with reduced levels of FLG degradation products and skin barrier function (Haftek et al., 2020). Furthermore, FLG metabolites, such as urocanic acid and pyrrolidone carboxylic acid, were shown to contribute to moisturization and maintenance of acidic pH of the SC. Both of these molecules may be crucial to epidermal barrier homoeostasis by regulating the activity of multiple enzymes which control desquamation, lipid synthesis and inflammation in AD skin (Meckfessel and Brandt, 2014; Moosbrugger-Martinz et al., 2022).

FLG deficiency may lead to impaired skin barrier function in AD through multiple pathways. It has been also shown in murine models, that the genetic modifications may affect microbial colonization (Kobayashi et al., 2015; Nakatsuji et al., 2016). However, FLG mutations have not been demonstrated in all of AD patients and also some patients with FLG mutations do not present dysbiosis of the microbiome (Kobayashi et al., 2015; Nakatsuji et al., 2016; Williams et al., 2020). Further studies seem to be necessary to clarify the relationship between the skin microbiome and FLG mutations. It is also clear that the FLG mutation is not the only and/or absolute factor in the etiopathogenesis of AD.

### The environmental factors associated with AD

Two hypotheses have been suggested for the development of AD lesions. The first one is the “inside-outside hypothesis”. This hypothesis is explained with epidermal barrier impairment in AD as a secondary result of the inflammatory response to irritants and allergens (Leung, 2000). On the other hand, the second one is the “outside-inside hypothesis” which favors that the xerosis (Denda et al., 1998), and the abnormal permeability of the barrier (Elias et al., 1999) or both may lead to AD lesions (Elias et al., 1999; Chamlin et al., 2002).

Microbial pathogens including bacteria, viruses and fungi may trigger AD and initiate allergic sensitization. These factors are modifiable and recognized by cell receptors. Thus, the skin immune cells are activated and secrete inflammatory cytokines causing the development of AD (Cork et al., 2009). Aeroallergens are also one of the AD triggers and include indoor aeroallergens such as house dust mite (HDM), pet dander, fur, cockroach, and mold, and outdoor aeroallergens such as tree, grass, and weed pollen (Werfel et al., 2015; Chong et al., 2022). HDM is a common sensitizing factor in pediatric AD patients and it has been also reported that children with a strong skin prick test reaction to HDM have more severe disease (Kutlu et al., 2013).

Various chemicals and irritants have been shown to influence AD, including soaps and detergents, as well as washing with hard water (Danby et al., 2018). Epidermal barrier dysfunction makes the skin vulnerable against these environmental factors, resulting in epidermal barrier damage. Detergents which contain irritating ingredients such as surfactants, may cause skin dryness, tightness and roughness, resulting in erythema and swelling (Ananthapadmanabhan et al., 2004). In AD patients, Callahan et al. showed lower thresholds to irritancy by sodium lauryl sulphate, which is a chemical agent in hand cleansers or shampoos (Callahan et al., 2013). Mechanical epidermal trauma could also exacerbate the symptoms of AD (Lee, 2020).

Climate is one of the associated factors with AD. It has been suggested that warm temperatures, high sun exposure or UV index, and higher humidity are associated with decreased AD prevalence, while low UV exposure, low temperatures and indoor heating may increase the risk of disease onset (Silverberg et al., 2013). However, higher temperatures and increased sun exposure may exacerbate the disease in some AD patients (Sargen et al., 2014; Kantor and Silverberg, 2017). These factors could be associated with the alterations in keratinocyte metabolism and immune dysregulation, as well as the degradation of FLG, which is affected by climate changes (Kantor and Silverberg et al., 2017). Vocks et al. showed that the temperature change from very cold (−17°C) to moderate (+18°C) was associated with decreased pruritus severity in AD patients (Vocks et al., 2001). Moreover, it has been suggested that people living in warmer climates may spend more time outdoors, thus they have more UV exposure, which may protect against AD. Contraversely, people living in warmer climates use less indoor heating, which may aggravate AD (Silverberg et al., 2013).

The relationship between birth season and AD has been investigated. Calov et al. (2020) found a significant association between AD and fall birth and winter birth when compared to spring birth. They suggested that the higher prevalance of AD in specific seasons could be explained by reduced ultraviolet radiation exposure, as well as increased air pollution (Calov et al., 2020) Air pollution is one of several factors that people are exposed to in daily life, and a contributor to AD. Air pollutants may arise from indoor and/or outdoor environments and they could enter the systemic circulation through penetration of the skin (Ahn, 2014). Rutter et al. found that the exposure to heavy traffic during the 12 months before measurement was significantly associated with eczema symptoms in children (Rutter et al., 2019) In contrast, Huls et al. showed no association between the traffic-related air pollution and AD in the general population (Huls et al., 2019). However, they suggested that the prevalence of childhood AD is correlated with oxidative stress and inflammation (Huls et al., 2018). They also reported that the risk scores from glutathione S-transferase P1, tumor necrosis factor, Toll-like receptor (TLR)-2, and TLR-4 single-nucleotide polymorphisms are associated with AD up to the age of 2 years (Huls et al., 2019; Ahn et al., 2020).

Exposure to certain foods may induce an immunological response in the skin and exacerbate the symptoms of some AD patients through allergic and non-allergic hypersensitivity reactions. It has been shown that more severe disease activity is correlated with increased frequency of food allergy (Burks et al., 1998; Eigenmann and Calza, 2000). Children with food allergies have positive skin tests and/or presence of serum IgE antibodies against particularly eggs, milk, wheat, soy and peanuts (Wassmann-Otto et al., 2018; Wollenberg et al., 2020). In a large population-based study, it has been reported that infants with AD are 6 times more likely to have egg allergy and 11 times more likely to have peanut allergy by 12 months than infants without AD at 12 months of age (Martin et al., 2014). However, restricted diet is not recommended for most AD patients (Rustad et al., 2022) since the possible tolerance of food allergies is being developed until the age of three (Akdis et al., 2006; Pelc et al., 2018). Elimination diet should be recommended in case a food is clearly identified as an exacerbarating factor (Papapostolou et al., 2022).

### The relationship between immune abnormalities, keratinocytes and cutaneous microbiome in AD

As the skin microbiome gains more and more interest as a key determinant of skin health, much effort is made to unravel the complex interplay of host cells and skin residents. In the last decade, it was shown in multiple studies that skin immunology and therefore also inflammatory processes are tightly and inevitably linked to the skin’s microbiota. Especially in the last few years a lot of evidence beginning to unravel the complex symbiosis between host and microorganisms was collected. The epidermis is colonized by bacteria, fungi, viruses and other microorganisms, and there is a complex interplay between host cells and the so-called commensals. From birth on, the skin of the new-born is colonized by microorganisms, with differences according to the mode of delivery (natural birth versus caesarean section). Based on environmental and individual factors, the microbiota stabilizes during the first years of life (Luna, 2020) Due to the importance of early microbial colonization, it can unsurprisingly be linked to the development of allergic diseases like food allergy or AD (Peroni et al., 2020). Therefore, human health is not only dependant on the actual microbial environment but also on the microbes that were encountered in the past, especially the first few years of life.

The cutaneous microbiome is important for both immune maturation and epidermal barrier function. There are many factors, including skin immune system, pH and water balance, the epidermal lipid composition and the expression of antimicrobial peptides, that contribute to the microbial balance in the epidermis (Zhang and Gallo, 2016; Wohlrab et al., 2018; Fölster-Holst, 2022). Because the skin’s microbiota (as well as microorganisms in other habitats) affects immunologic maturation, characteristic changes in skin colonization like the overrepresentation of pathogenic bacteria or the lack of contact to “good” ones in early life may have consequences for inflammatory skin diseases and might be even involved in their pathogenesis. For instance, this has been demonstrated for AD. Meylan and others found that *S. aureus* colonization in infancy was positively associated with the development of AD, and this colonization preceded disease onset (Meylan et al., 2017). In contrast, the presence of *Staphylococcus* hominis at age 3 months tended to be negatively associated with AD development, underscoring that different bacteria exert different effects to the skin and its immune system (Meylan et al., 2017). Moreover, during AD flares, the variety of skin bacteria colonization alters and *S. aureus* colonization increased and S. epidermidis decreased (Bjerre et al., 2017). On the contrary, S. epidermidis and species of *Streptococcus*, Corynebacterium and Propionibacterium increased during the remission phases (Kong et al., 2012). However, it has been reported that antibacterial agents against *S. aureus* are not superior to other non-antimicrobial treatments in AD (Bath-Hextall FJ et al., 2010). *S. aureus* may not be a major pathogenic factor in the etiopatogenesis of AD, nevertheless the presence of S. aureus could be associated with disease severity (Moosbrugger-Martinz et al., 2021). In adult patients with severe AD during disease flare, increased levels of *S. aureus* in lesional skin have been shown (Clausen et al., 2018; Smits et al., 2020). Moreover, *S. aureus* colonization in non-lesional skin and even twice more in lesional skin have been shown in patients with AD (Baker, 2006; Totté et al., 2016; Nowicka et al., 2022). However, it has not been found in all AD patients (Paller et al., 2019b) and it is not certain whether *S. aureus* promotes the inflammation in AD or whether the inflammation is the reason for the presence of *S. aureus* due to an impaired skin barrier. *S. aureus* secretes toxins and superantigens and stimulates the activation of T cells, thus contributes to skin inflammation in patients with AD (Schlievert et al., 2010). In support of this, Skop et al. suggested that the application of Staphylococcal enterotoxin B to non-lesional skin may induce eczematous lesions (Skov et al., 2000). On the other hand, the colonization with some “good” bacteria on the skin may have protective effects in AD (Cho et al., 2001). In patients with AD and Netherton syndrome, increased S. epidermidis and S. hominis in the post flare phase have been shown (Byrd et al., 2017; Moosbrugger-Martinz et al., 2021). Some strains of S. epidermidis may improve innate immunity and protect the skin against infection with pathogens by activating IL-17- expressing CD8^+^ T cells (Prescott et al., 2017).

The present microbes and their metabolites also prime the maturation of the host immune system. Innate as well as adaptive immune responses are different in neonates compared to adults. In order to ensure tolerance to self- and foreign antigens, neonates are less prone to inflammation, which is crucial for the rapidly developing tissues (Paller et al., 2019b). During this educational period for the host immune system, the foundation for health or disease in later life could be established, as demonstrated in animal models, e.g., for inflammatory bowel disease and asthma (Gensollen et al., 2016). For the skin, this was shown with the commensal *Staphylococcus* epidermidis, which is considered a beneficial bacterium in this tissue. Adult immunologic responses to this commensal were different in mice according to the time of introduction of *S. epidermidis*. Exposure in early life circumvented inflammation induced by contact in adult animals, and this effect was attributed to the development of regulatory T-cells in answer to early exposure (Scharschmidt et al., 2015).

Keratinocytes are directly involved in the regulation of commensal-specific T cells. Epidermal accumulation of lymphocytes is a phenomenon induced by the microbiota, with type 1 and type 17 cells present in these clusters. Commensal induced Th1 cells thereby are regulated by MHC class II expressing keratinocytes in an IL-22 dependent fashion (Tamoutounour et al., 2019). The epidermal microbiota seems to be more individual than the dermal one, which shows less inter-individual variability (Bay et al., 2020). Host cells like keratinocytes establish an environment in favour of beneficial microbes by secreting specific antimicrobial components including innate cytokines, pathogen associated molecular pattern (PAMP) molecules, the inflammasome, and AMPs, targeting unwanted microorganisms, while providing nutrition for those who can metabolize special lipids (Nakatsuji et al., 2010; Dahlhoff et al., 2016).

Next to specific antimicrobial substances, bacteria are influenced by other host signals whose primary function is the communication between host cells. For example, it was found that neuropeptides like substance P exert effects on the virulence of bacteria, thereby contributing to microbiota homeostasis (N’Diaye et al., 2017).

The high diversity of the gut microbiome affects the immunity of the whole body, including skin. It improves the levels of regulatory T cells and short-chain fatty acids (Belkaid and Hand, 2014). On the other hand, an imbalance of the gut microbiome and increased levels of noxious microorganisms lead to secondary skin infections and immune-related diseases including AD (Belkaid and Hand, 2014). Infants with a diverse gut microbiome have a lower risk of development of AD (Ihekweazu and Versalovic, 2018; Park et al., 2021). In addition to this, infants with AD are more often colonized by *Clostridium difficile* and *Escherichia coli* than infants without AD (Penders et al., 2007). The priming of the cutaneous immune system enables the mature tissue to select for beneficial microorganisms, which at the same time prevent the outgrowth of pathogenic species. By competing for space and nutrition and by specific antagonistic mechanisms, commensal bacteria protect their respective niche. This delicately balanced homeostasis is disrupted in different disease settings. Dysbiosis, which means an adversely altered microbiota, can trigger inflammatory processes, e.g., by inducing IL-1α release by keratinocytes (Archer et al., 2019). Archer and others demonstrated in a filaggrin deficient mouse model, that skin injury and dysbiosis resulted in chronic inflammation in the animals, and that keratinocyte-derived IL-1α was the driver of this development. This suggests that skin microbiome modulation could be beneficial for AD patients.

Several regulators and signalling pathways were found to be involved in the skin-commensal crosstalk and the cutaneous microbiota homeostasis. In mice, dendritic epidermal T cells are regulated by the G protein coupled receptor 15 (GPR15) (Sezin et al., 2021). Gpr15−/− mice exhibit a severe deficiency in this cell type, with an overall reduced number of T cells in the epidermis, but not the dermis. The expression of GPR15 on T cells in turn is influenced by the skin as well as the gut microbiome (Kim et al., 2013; Jacob et al., 2020), and knock-out animals show alterations of their cutaneous microbiomes compared to wildtype littermates. Interestingly, GPR15 expression in human CD4^+^ cells is regulated by aryl hydrocarbon receptor (Ahr) (McAleer et al., 2018), and since dendritic epidermal T cells depend on Ahr signalling (Kadow et al., 2011), Sezin et al. speculated about GPR15 as a downstream target of Ahr (Sezin et al., 2021). Furthermore, keratinocyte Ahr is essential for barrier integrity in murine skin (Haas et al., 2016), demonstrating the importance of the Ahr pathway in different cell types in the epidermis. Just like Gpr15−/− mice, Ahr-deficient mice demonstrate an altered, more variable and probably more unstable microbiome (Haas et al., 2016). Ahr is activated by indole-3-aldehyde, a microbial tryptophan metabolite, and Ahr activation leads to production of anti-inflammatory IL-10 by Langerhans cells and subsequently inhibited CD4^+^ T cell proliferation (Liu et al., 2020). In addition, Ahr signalling in keratinocytes is influenced by the microbiota, with consequences for skin barrier function and repair (Uberoi et al., 2021).

A further player in the dialogue between skin immune cells and microbiota is the transcription factor JunB, which is expressed by keratinocytes. Mice deficient in epidermal JunB exhibit an AD-like phenotype, including proneness to spontaneous skin colonization by *S. aureus*. JunB negatively regulates MyD88 in keratinocytes, upon loss an inflammatory response cascade was reported. As inflammation was exacerbated in Rag1−/− mice, it was concluded that the adaptive immune system, presumably due to the production of IL-17A by T cells, is necessary to prevent *S. aureus* infection (Uluçkan et al., 2019).

Another example of the complexity of the regulation of epithelial cells, immune cells and commensals is the homeostatic control of sebaceous glands by innate lymphoid cells residing in hair follicles. Innate lymphoid cells express mediators that restrict sebocyte growth, thereby regulating the expression of sebaceous gland derived antimicrobial lipids. In this way, the commensal bacteria equilibrium is maintained (Kobayashi and Nagao, 2019). Hair follicles themselves rely on the endopeptidase ADAM10-Notch signalling axis, with a disruption in this pathway resulting in skin dysbiosis and destruction of hair follicles (Sakamoto et al., 2021).

While the interactions between host skin cells and commensals are already a complex and delicately balanced system, another layer of complexity is added when considering the skin as part of the whole human body. Not only keratinocytes and epidermal immune cells receive signals from within the body, so do the microorganisms which stand in contact with other microbial habitats from their host organism. Especially the gut as the most heavily colonized region is thought to act as a central signalling node for all peripheral microbial communities (Martínez et al., 2021). The cutaneous microbiome therefore is not only important for immune maturation and regulation but is directly involved in epidermal function. In conclusion, future work should be directed at further unravelling the network of interactions and molecular signalling mechanisms that include keratinocytes, skin immune cells and microorganisms.

### Cutaneous inflammation, AD and cancer risk

The relationship between inflammatory skin disorders including AD, and cancer has been investigated (Zhu et al., 2022; Wan et al., 2023). AD has been found to be significantly correlated with an increased risk of non melanoma skin cancer (NMSC) (Zhu et al., 2022). In line with this, in another study, a greater risk of NMSC in children with mild AD was reported, however, a lower risk of melanoma in children with moderate AD was found (Vittrup, 2023). Further, an increased risk of lymphoma among children with severe AD has been found (Vittrup, 2023). In adults, a slightly increased risk of haematological malignancy, aslightly higher skin cancer risk and lower risk of solid organ malignancy has been reported (Vittrup, 2023). The authors suggested that although there is no overall association between AD and malignancy, AD may have heterogeneous effects by cancer subtype (Vittrup, 2023).

Although the mechanism of skin cancer in AD patients is not certain, skin cancer progression has been associated with the dysregulation of microbiome (Woo et al., 2022). Furthermore, it can be suggested that the increased risk of skin cancer in patients with AD may be a long-term side effect of phototherapy treatment. However, the reduced risk of melanoma contradicts this hypothesis (Vittrup, 2023). Moreover, in recents studies, no strong overall malignancy risk in AD but a possible increased lymphoma risk in patients with severe AD (Wan et al., 2023), particularly NHL, that increased with eczema severity (Mansfield et al., 2020), has been reported.

On the other hand, decreased risk of malignancy in the esophagus, stomach, colorectum, and liver, has been found in patients with allergic diseases including allergic rhinitis, asthma, and AD (Choi et al., 2023). This decreased risk in GI cancers in AD patients could be explained by the immunosurveillance hypothesis. According to this theory, excessive stimulation of T-helper cell type II immune response and other immune cells such as mast cells, natural killer cells and eosinophils can prevent the onset of cancer by detecting and destroying the damaged cells before the onset of carcinogenesis (Ji et al., 2009; Choi et al., 2023). On the contrary, it is known that a Th2-dominant environment downregulates tumor immunity (Morimura et al., 2021). However, Morimura et al., suggested that a high level of CCL17, known as thymus and activation-regulated chemokine, may work as a “safety-net” to reduce the risk of malignant tumors and positively contributes to tumor immunity via decreasing myeloid-derived suppressor cells in the Th2-dominant environment in AD patients (Morimura et al., 2021). This may be one of the explanations for the normal incidence of cancer among patients with AD, regardless of the Th2-dominant environment. However, it is clear that the incidence of cancer in patients with AD needs further investigation.

### Therapeutic options for AD and their effects on the immunological and structural epidermal barrier dysfunction and skin microbiome

AD presents quite heterogeneously, both clinically and immunologically (Czarnowicki et al., 2019). There are various treatment options for AD according to age, the severity of pruritus, involved body-surface area and the clinical stage of the disease (mild, moderate, or severe) (Sidbury et al., 2014; Wollenberg et al., 2018). A multi-therapeutic approach is essential in the management of AD. Short-term management aims to control symptoms in the periods of exacerbation of AD, while long-term treatment aims to prevent from new lesions and prolong the time between flares (Boulos and Yan, 2018). The most important aim of AD treatment is disease prevention due to potential toxicity of immunosuppressive therapies (Diaz and Guttman-Yassky, 2019).

Although it is not possible to mention all of the drugs marketed or being tested, there are approved topical and systemic treatment options available in the treatment of AD. Basic therapy contributes to barrier function through improving skin hydration and regeneration of intercellular lipid lamellae and is always required in addition to other topical and systemic therapeutics (Staubach and Lunter, 2014). Moisturizers are used both for the therapy and prevention of AD, providing repair to the epidermal lipid matrix (Elias et al., 2019). Moisturizers also reduce the use of inflammatory products and improve epidermal barrier function (Loden, 2003; Szczepanowska et al., 2008; Czarnowicki et al., 2016; Giam et al., 2016). In premature newborns, moisturizers also decrease bacterial colonization, TEWL and disease severity (Nopper et al., 1996; Darmstadt et al., 2007). Additionally, petrolatum increases the expression of FLG and loricrin and also induces upregulation of major AMPs such as LL-37, lipocalin 2 and peptidase inhibitor 3 (Czarnowicki et al., 2016). Moreover, although there are opposing views, it has been reported that early moisturizing in high-risk newborns alters the skin microbiome and pH levels (Glatz et al., 2015).

In 1991, wet-wrap dressing was applied to pediatric AD patients for the first time (Goodyear et al., 1991). For such dressings, the patient takes a bath in warm water and dries off the water, after this, the lesions are covered with wet gauze. This wet material is then covered with a second layer of dry material for a certain time. Wet-wrap dressing may also be applied with moisturizer or weak topical corticosteroids according to the clinical findings of the disease (Twitchen and Lowe, 1998). Wet-wrap therapy is associated with improvement of epidermal barrier function in patients with AD. Lee et al. (2007) showed that wet-wrap dressing is associated with decreased SCORAD, increased epidermal water content, and decreased TEWL. However, they observed no change in keratinocyte differentiation and calcium ion gradient with wet-wrap dressing (Lee et al., 2007).

Application of topical corticosteroids (TCSs) remains to be the mainstay in the treatment of AD. TCSs have anti-inflammatory, anti-proliferative, and immunosuppressive effects which contribute to the treatment of AD (Del Rosso and Friedlander, 2005). Glucocorticoids show suppressive effects against neutrophils, monocytes, lymphocytes, Langerhans cells and cytokines such as IL-1α, IL-1β, IL-2, tumor necrosis factor, and granulocyte-monocyte colony stimulating factor (Del Rosso and Friedlander, 2005). In contrast to the beneficial effects of steroids, they also inhibit the synthesis of cholesterol, ceramides, and free fatty acids and disrupt the anti-bacterial function of the epidermis (Pelc et al., 2018). Long-term use of TCSs leads to various side effects including skin atrophy, striae distensae, teleangiectasia, and impaired skin barrier (Schoepe et al., 2006; Shlivko et al., 2014). Therefore, topical corticosteroids should be used only for a certain period of time and in appropriate amounts.

There are also other topical therapeutic options such as topical calcineurin inhibitors (TCIs) and topical phosphodiesterase 4 (PDE4) inhibitors with advantages of minimal side effects and possible long-term use (Kim et al., 2020; Freitas et al., 2022). TCIs inhibit the transcription of proinflammatory cytokine genes, including IL-2 (Bornhovd et al., 2001). TCIs are favorable particularly in skin folds and at the face, which are highly sensitive areas (Simpson, 2010). Jensen et al. reported improvement in all epidermal barrier parameters including TEWL in AD patients which were treated with both a topical steroid (betamethazone valerate) and a topical calcineurin inhibitor (pimecrolimus). Although both treatments normalized epidermal differentiation and reduced epidermal hyperproliferation, betamethazone valerate has been found to be more effective in reducing clinical symptoms and epidermal proliferation, however it induced epidermal thinning unlike pimecrolimus (Jensen et al., 2009). Their alternate use may be an option in AD lesions.

Crisaborole ointment 2% is a nonsteroidal PDE4 inhibitor for the treatment of mild-to-moderate AD. The inhibition of PDE4 may decrease the inflammatory processes associated with AD without significant serious adverse event incidences. PDE4 inhibitors reduce the occurrence of AD exacerbation, however they have a statistically significant risk of producing pain (Martín-Santiago et al., 2022). Moreover, roflumilast (Calverley et al., 2007) and apremilast (Papp et al., 2013) have several adverse effects after systemic administration, but topical application has been found to decrease exposure and minimize adverse effects including burning of skin, pruritus and skin infections (Ashcroft et al., 2005; Broeders et al., 2016; Abed and Pawliczak, 2019).

The gut-skin-axis is involved in several dermatological diseases including AD (De Pessemier et al., 2021). In inflammatory skin diseases like AD or psoriasis, increasing interest is directed at the microbial skin component as therapeutic target. Influencing skin commensals and/or pathogenic bacteria like *S. aureus* with probiotics could happen via the gut-skin axis with selected bacteria (Szántó et al., 2019). Topical or oral probiotics may have some beneficial effects on AD symptoms associated with gut microbiome dysbiosis, including the alteration of the abundance of skin commensals and/or pathogenic bacteria like *S. aureus* (Szántó et al., 2019; Fang et al., 2020). It has been reported that *Lactobacillus* johnsonii caused significant improvement in skin symptoms of AD patients (Szántó et al., 2019) and also *Lactobacillus* plantarum significantly decreased SCORAD index and influencds the gut microbiota composition (Fang et al., 2020). Other approachs are even more direct and are supposed to modulate the skin microbiome by immediate application of specific bacteraial strains to the skin. Isolation of Roseomonas mucosa strains from healthy volunteers and transplantation to adult and peadiatric AD patients was successfully performed and led to reduced *S. aureus* colonization (Myles et al., 2018; Myles et al., 2020). Furthermore, SCORAD and Eczema Area and Severity Index (EASI) decreased and patients needed less glucocorticoid treatment. The authors found that Roseomonas mucosa produced beneficial sphingolipids and induced TNFR2-mediated epithelial repair mechanisms (Myles et al., 2020).

In another study aiming at the decrease of *S. aureus* colonization, autologous transplantation of specific bacterial strains like *S. epidermidis* and *S. hominis* after screening for antimicrobial activity achieved good results in AD patients and demonstrated the importance of skin commensals for AD disease pathology (Nakatsuji et al., 2017). In a corresponding phase 1 trial, a *S. hominis* strain isolated from healthy human skin was used as bacteriotherapy for AD and decreased *S. aureus* (Nakatsuji et al., 2021). Although eczema severity was not significantly changed over all patients, a specific subgroup seemed to especially benefit from the *S. hominis* treatment. This finding highlights that when it comes to the microbiome and its role in diseases, there will be huge differences between patients and individualized therapy approaches are or will be warranted.

In recent years, new therapeutic options such as biologic drugs have gained more importance with better understanding of AD pathogenesis (Kim et al., 2020; Freitas et al., 2022). Dupilumab is a human monoclonal antibody against IL-4 receptor α. Berdyshev et al. investigated the role of dupilumab in the regulation of skin barrier structure and function. They reported that blocking IL-4/IL-13 signalling with dupilumab decreased the TEWL in AD lesions, normalized the lipid composition and increase the ceramide chain length in lesional as well as non-lesional SC of AD patients (Berdyshev et al., 2022). Recently, the effect of dupilumab on *S. aureus* colonization and microbial diversity of the skin has been investigated (Callewaert et al., 2020). During dupilumab therapy, Callewaert et al. showed in both, lesional and non-lesional skin, increased microbial diversity and decreased S. aureus colonization, which was correlated with clinical improvement of AD (Callewaert et al., 2020). Dupilumab seems to affect many factors such as immunological markers, lipid composition and microbial colonization in the etiopathogenesis of the AD.

With immunological discoveries in the etiopathogenesis of AD, new biological therapies are gaining importance in the treatment of the disease. IL-13 is a potential therapeutic target for patients with AD (Zhang et al., 2022). In AD patients, increased expression of IL-13 in lesional tissues and elevated serum IL-13 levels have been found when compared to healthy controls (Tazawa et al., 2004). Moreover, it has been reported that an elevated IL-13 level is positively correlated with AD disease severity (Tazawa et al., 2004; Ungar et al., 2017). Tralokinumab is a human IgG4 monoclonal antibody, preventing IL-13 from binding to both IL-13Rα1 and IL-13Rα2 (Popovic et al., 2017). In a recent report from 2,285 patients, tralokinumab has been found to be a well-tolerated agent both in combination with TCS and as monotherapy, with long-term use up to 52 weeks for moderate-to-severe AD (Simpson et al., 2022). Lebrikizumab, which is also one of the new biological therapies, is a selective monoclonal antibody that targets IL-13. IL-13 plays an important role in multiple itch pathways and may contribute to the persistence of chronic itch in AD. The improvement of chronic itch in AD patients by lebrikizumab seems to be related to neuronal effects via IL-13 inhibition (Miron et al., 2022). A potential advantage of IL13 inhibitors is that the conjunctivitis, which is a common side effect of dupilumab (up to 22% in clinical trials), is less frequent under lebrikizumab (6.3%) and tralokinumab (6.2%) (Akinlade et al., 2019; Zhang et al., 2022).

Nemolizumab is a subcutaneously administered humanized monoclonal antibody against IL-31 receptor A. IL-31 blockage shows a direct effect against pruritus in AD patients (Nemoto et al., 2016; Oyama et al., 2018). Nemolizumab may also improve skin barrier function (Feld et al., 2016; Singh et al., 2016) and reduce the overall severity of AD (Liang et al., 2022). In a recent stıdy, it has been reported that nemolizumab achieves TIMEACLIR-Itch (MEAningful CLInical Response for itch reduction) more quickly than anti-IL-4 or -IL-4/13 agents (Lin et al., 2022). In 2022, nemolizumab has been approved in Japan for adults and children above the age of 13 years in the treatment of itch associated with AD, refractory to current treatments (Maruho, 2022). Additionally, numerous clinical trials are ongoing which are investigating the efficacy of nemolizumab in AD (Keam, 2022).

Topical and oral JAKi are potential treatment options which significantly improve the clinical symptoms of AD patients with unsatisfactory response to conventional therapeutics (Honstein and Werfel, 2020). IL-4, IL-13, and IL-31 are the major cytokines which influence AD pathogenesis via the JAK-STAT signalling pathway. Systemic JAKi including baricitinib (JAK1/2i), upadacitinib (JAK1i) and abrocitinib (JAK1i) are current treatment options in moderate to severe AD (Klein et al., 2022).

Ruxolitinib is also a selective JAK1/2 inhibitor available in both topical and oral administration options. Ruxolitinib 1.5% topical cream is the first topical JAKi, approved by US Food and Drug Administration (FDA) in patients (≥12 years) with mild-to-moderate AD (Owji et al., 2022). It has been reported that ruxolitinib and delgocitinib (pan JAKi) significantly improve pruritus and EASI in patients with moderate to severe AD (Kim et al., 2020; Nakagawa et al., 2020). In patients with mild to moderate AD with 2%–20% body surface area involvement, the efficacy of tofacitinib, which is a topical JAK 1/3 inhibitor, has also been reported (Bissonnette et al., 2016). In a recent meta-analysis, it has been found that particularly tofacitinib 2% has superior Investigator’s Global Assessment response over other included JAKi and PDE4 inhibitors, followed by ruxolitinib 1.5% and delgocitinib 3% (Zhang et al., 2021). However, it is clear that more studies comparing the effect of topical and/or biological agents in AD patients, are needed.

## 2 Conclusion

AD is a chronic inflammatory skin disease in which many factors such as immunological and structural epidermal barrier dysfunction, immune abnormalities, lipid alterations, FLG mutations and skin microbiome alterations are involved in its etiopathogenesis. Although much progress has been made regarding the pathophysiology of AD and its clinical manifestations in adults and children, there are still many points that need to be clarified including unclear, complex molecular and cellular mechanism and metabolics, and also the specific mechanism of the alterations of lipid compositions.

Although topical corticosteroids are the cornerstone of AD treatment, new biological therapies also achieve promising results. However, it is also necessary to monitor the long-term effects of biological treatments. With new discoveries in the etiopathogenesis of AD, the symptoms of the disease could be controlled more effectively with new targeted therapies.
